# Crystal Structure of the P Pilus Rod Subunit PapA

**DOI:** 10.1371/journal.ppat.0030073

**Published:** 2007-05-18

**Authors:** Denis Verger, Esther Bullitt, Scott J Hultgren, Gabriel Waksman

**Affiliations:** 1 Institute of Structural Molecular Biology, University College London and Birkbeck College, London, United Kingdom; 2 Department of Physiology and Biophysics, Boston University School of Medicine, Boston, Massachusetts, United States of America; 3 Department of Molecular Microbiology, Washington University School of Medicine, St. Louis, Missouri, United States of America; Harvard Medical School, United States of America

## Abstract

P pili are important adhesive fibres involved in kidney infection by uropathogenic Escherichia coli strains. P pili are assembled by the conserved chaperone–usher pathway, which involves the PapD chaperone and the PapC usher. During pilus assembly, subunits are incorporated into the growing fiber via the donor–strand exchange (DSE) mechanism, whereby the chaperone's G_1_ β-strand that complements the incomplete immunoglobulin-fold of each subunit is displaced by the N-terminal extension (Nte) of an incoming subunit. P pili comprise a helical rod, a tip fibrillum, and an adhesin at the distal end. PapA is the rod subunit and is assembled into a superhelical right-handed structure. Here, we have solved the structure of a ternary complex of PapD bound to PapA through donor–strand complementation, itself bound to another PapA subunit through DSE. This structure provides insight into the structural basis of the DSE reaction involving this important pilus subunit. Using gel filtration chromatography and electron microscopy on a number of PapA Nte mutants, we establish that PapA differs in its mode of assembly compared with other Pap subunits, involving a much larger Nte that encompasses not only the DSE region of the Nte but also the region N-terminal to it.

## Author Summary

Bacterial adhesion to a host is a crucial step that determines the onset of bacterial infection. It is mediated through recognition of a receptor on the host cell surface by a protein called an adhesin displayed on the surface of the bacterium. Many adhesins are displayed at the tip of specialized organelles called pili, some of which are assembled by the ubiquitous chaperone–usher pathway. In this pathway, each pilus subunit is assisted in folding by a chaperone. The resulting chaperone–subunit complex is targeted to a pore located in the outer membrane, called the usher, that serves as assembly platform. There, pilus subunits dissociate from the chaperone and polymerize, resulting in a surface organelle, the pilus, that protrudes out of the usher. Here, we have determined the structure of the major subunit of the P pilus, PapA. The P pilus, produced in uropathogenic *Escherichia coli,* displays the adhesin PapG responsible for targeting the bacterium to the kidney epithelium. We have determined the structure of PapA either bound to its cognate chaperone, PapD, or bound to another PapA subunit. These structures provide a view of PapA before and after its assembly in the pilus and shed light on the mechanism of PapA assembly.

## Introduction

Urinary tract infections, which include infections of the bladder (cystitis) and kidney (pyelonephritis), are some of the most common bacterial infections. These infections are caused mainly by uropathogenic Escherichia coli [1]. Once uropathogenic E. coli is introduced, survival and persistence of these bacteria in the urinary tract require a specific set of virulence factors, including the expression of type P pili. P pili are specifically required for the ability of uropathogenic E. coli to bind Gal-α (1–4)-Gal moieties in human kidney cells and cause pyelonephritis [2,3]. P pili are encoded by the *pap* gene cluster and are assembled via the highly conserved chaperone–usher pathway, involving the periplasmic immunoglobulin (Ig)–like chaperone PapD and an outer membrane usher PapC [4,5]. P pili consist of six subunits making up a composite fiber with a short tip fibrillum composed of the PapE subunit joined to a more rigid helical rod composed of the PapA subunit [6,7]. PapG is the adhesin at the end of a tip fibrillum; PapK and PapF are adaptor subunits between the PapA rod and the PapE fibrillum and between the PapE fibrillum and the PapG adhesin, respectively; finally, PapH terminates P pilus formation [8,9]. The PapA rod is formed by more than 1,000 PapA molecules assembled in a right-handed helical manner, with 3.3 molecules per turn [6,10].

**Figure 1:**
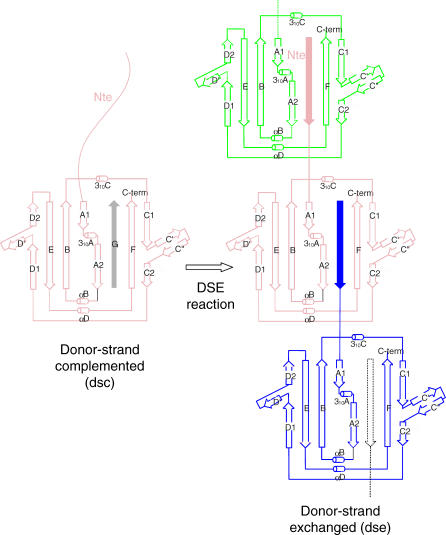
Schematic Diagram Illustrating the Processes of DSC and DSE In this diagram, β-strands and helices are represented as arrows and cylinders, respectively. At left, a topology diagram of a subunit bound to the chaperone is shown in pink. The Nte is shown as a disordered region at the N-terminus of strand A1. In the chaperone, only the G_1_ strand is shown in gray: it complements the fold of the subunit by inserting between strands A2 and F, providing *in trans* the missing G strand. Note that the complementing G_1_ strand runs parallel to strand F, thereby reconstituting an atypical Ig-fold for the subunit. The donor–strand-complemented subunit is referred to in the text as “dsc”. At right, the same subunit in pink is shown in the pilus. Its Nte is now inserted between strand A2 and F of the subunit in green. Its groove is now filled with the Nte of the subsequent subunit in dark blue. The subunit is referred to in the text as “dse”.

All pilin subunits adopt an Ig-like fold but lack the seventh, C-terminal G β-strand, thus producing a large hydrophobic groove on the side of the protein (Figure 1 and [11,12]). In a process called donor–strand complementation (DSC), the G_1_ β-strand of PapD inserts a conserved motif of three alternating hydrophobic residues (called the P1 to P3 residues) plus N101 (P4 residue) into four binding pockets in the hydrophobic groove of the pilus subunits (P1 to P4 binding pockets). The G_1_ strand provides the structural information lacking in the pilus subunit by completing its Ig fold [11–13]. Pilus subunit assembly proceeds through a noncovalent polymerization process called donor–strand exchange (DSE; Figure 1). All subunits, except for the adhesin, possess an N-terminal extension (Nte) peptide of 11 (PapK), 12 (PapE and PapF), 19 (PapA), or 33 (PapH) residues (Figures 1 and S1) that is disordered and not part of their Ig-like structure. The Nte comprises a highly conserved array of alternating hydrophobic residues, called the P2 to P5 residues [14,15]. This array constitutes the DSE region of the Nte (see Figure S1 for location of this DSE region). As chaperone–subunit complexes are differentially targeted to the usher [16,17], each subunit donates its Nte to complete the Ig-fold of the subunit previously assembled by inserting its P2–P5 residues into the corresponding P2–P5 binding pockets, thus first displacing and then replacing the chaperone G_1_ strand in the groove of the previously assembled pilus subunit [18–21]. This process occurs through a zip-in–zip-out process whereby the DSE reaction is initiated by the insertion of the P5 residue of the Nte of one subunit into the P5 pocket of the groove of the other [21]. This binding event leads to the formation of a transient ternary complex, the formation of which is essential for the DSE reaction to proceed. Insertion of the Nte of PapH into the groove of a PapA subunit terminates pilus biogenesis because PapH lacks a P5 pocket and thus cannot provide the initiator-binding event required for the exchange reaction with another subunit [9].

Recently, in a departure from the more conventional model described above, Mu et al. [22] suggested, based on electron microscopy and image reconstruction of the PapA rod, that the DSE region of the Nte of PapA is not involved in DSE, but instead the region of the Nte N-terminal to the DSE region is involved in the process. Interestingly, the Nte of PapA is longer than the Nte of most Pap subunits except PapH (Figure S1), and thus the model proposed by Mu et al. [22] could potentially explain why residues N-terminal to the DSE region would be required in the process of P pilus biogenesis. This is explored further in this report, in which we describe the structures of a binary complex of PapD bound to PapA and of a ternary complex containing the chaperone PapD and two PapA subunits. In this ternary complex, PapD is bound to PapA through DSC, and this subunit is itself bound to another PapA subunit through DSE. These structures are used as a basis for a detailed mutational study dissecting the requirements for PapA polymer formation.

## Results/Discussion

### Crystal Structures of PapD/PapA_Ntd1_G15N_ and PapD/(PapA_Ntd1_G15N_T101L_)_2_

In order to investigate the PapD/PapA and PapA/PapA interactions within the P pilus rod, the first structure which we solved was that of PapD/PapA, where two mutations were introduced in the *papA* gene. This was necessary because PapA in purified wild-type PapD/PapA complexes tends to spontaneously polymerize, and self-polymerization of PapA has prevented structural studies. As PapA, like any other pilus subunits, polymerizes through DSE, mutating residues in the Nte was an obvious starting point to obtain a PapA mutant unable to polymerize. We first deleted the entire Nte (residues 2 to 19; PapA_Ntd2_; Figure S1), but coexpression of such mutants with PapD did not result in a material amenable to purification (unpublished data). Thus, a second mutant was designed that conserved the entire Nte but introduced an Asn at position 15. Position 15, a Gly residue in the wild-type Nte of PapA, locates in the DSE region of the Nte and is strictly conserved among all Pap Ntes (Figure S1). G15 is indeed required because, after DSE, it lies in the P4 pocket of the subunit's grooves, which, in this region and in all Pap subunits, contains a bulky phenylalanine or a tyrosine (F152 in PapA, Y146 in PapK, F138 in PapE, F137 in PapF, and Y162 in PapH) [11,18]. We thus mutated G15 to N; however, PapA_G15N_ in complex with PapD also undergoes spontaneous polymerization (Figure S2A, top panel). Thus, in addition to the G15N mutation, a mutant where residues 2 to 8 were deleted was next constructed. The deleted region is just N-terminal to the DSE region of the Nte of PapA. This mutant (PapA_Ntd1_G15N_) did not undergo spontaneous polymerization, and formed a stable complex with PapD (Figure S2A, middle panel). This experiment indicates that, as suggested by the electron microscopy study of Mu et al. [22], the region of the Nte N-terminal to the DSE region is involved in PapA/PapA interaction. Crystals of PapD/PapA_Ntd1_G15N_ diffracted to a resolution of 2.6 Å, and the structure was solved by molecular replacement using the PapD/PapK structure as a search model. This structure is very similar to the already known PapD/PapK, PapD/PapE_Ntd_, or PapD/PapH_Ntd1_ structures [9,11,18]. Like PapK, PapE, or PapH, PapA lacks strand G of its Ig-fold; PapD complements this by donating its G_1_ strand.

**Figure 2:**
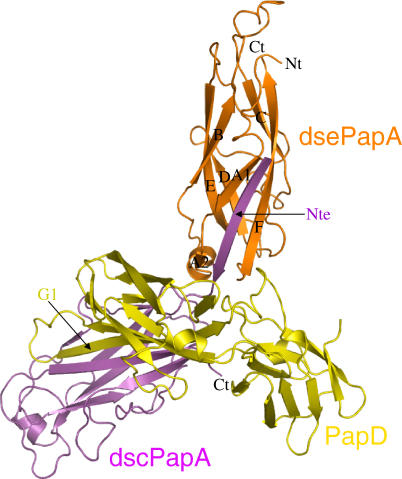
Crystal Structure of PapD/(PapA_Ntd1_G15N_T101L_)_2_ Overall 3-D structure of the PapD/(PapA_Ntd1_G15N_T101L_)_2_ complex: PapD (yellow), dscPapA (purple), and dsePapA (orange) are shown in ribbon representation with β-strand indicated as arrows and α-helices indicated as cylinders. The β-strands of dsePapA are labeled, as are the PapD G_1_ strand and the dscPapA Nte. Figures 2–5 were made using PyMol (http://www.pymol.org).

By itself, the PapD/PapA_Ntd1_G15N_ structure is not very informative. However, by investigating a different PapA mutant with the additional mutation T101L, we obtained crystals of a complex containing one PapD molecule and two PapA_Ntd1_G15N_T101L_ molecules. This T101L mutant was initially designed to fill in the PapA P5 pocket. Indeed, as explained in the Introduction, all Pap subunits, except PapH, have a clear P5 pocket, which serves as an initiator point, the occupation of which triggers the DSE reaction [9,21]. Thus, the PapA_Ntd1_G15N_T101L_ was made to test the possibility that by filling its P5 pocket, PapA would become more like PapH in being unable to undergo DSE. As shown in Figure S2B, indeed, PapA_Ntd1_G15N_T101L_ has reduced DSE activity compared with that of PapA_Ntd1_G15N_. However, this T101L mutation had the additional unexpected consequence of stabilizing a complex containing a 1:2 molar ratio of PapD and PapA (Figure S2A, lower panel). PapD/(PapA_Ntd1_G15N_T101L_)_2_ was thus purified. Crystals were produced diffracting to 2.5 Å resolution. The structure of this ternary complex is shown in Figure 2. It clearly shows one PapA molecule bound to PapD through DSC in a complex very similar to PapD/PapA_Ntd1_G15N_; however, this time, the Nte of the donor–strand-complemented PapA molecule is bound to the groove of another PapA molecule, and thus this ternary complex crystal structure provides a snapshot of PapA before and after DSE. In that respect, the PapD/(PapA_Ntd1_G15N_T101L_)_2_ complex is similar to the one obtained by Zavialov et al. for the Caf system [19].

### Comparison between the Two PapA Molecules in the PapD/(PapA_Ntd1_G15N_T101L_)_2_ Structure

**Figure 3:**
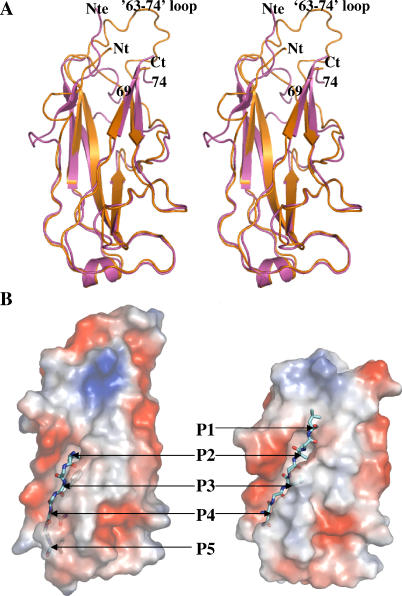
Comparison of dsePapA and dscPapA in the PapD/(PapA_Ntd1_G15N_T101L_)_2_ Structure (A) Stereo ribbon diagram of dsePapA (orange) and dscPapA (purple). The N- and C-termini of both PapA molecules are indicated, as well as the “63–74” loop in dsePapA. In dscPapA, this loop is missing residues 70–73; thus, residues 69 and 74 are labeled. (B) Surface representation of dsePapA (left) and dscPapA (right). Residues of the chaperone G_1_ strand as well as dscPapA Nte are shown in stick representation color-coded in red, dark blue, and cyan for oxygen, nitrogen, and carbon atoms, respectively. For clarity, only side chains of the residues interacting in the P1 to P4 (right) or P2 to P5 (left) pockets are shown.

Figure 3A shows a superimposition of the two PapA molecules in the PapD/(PapA_Ntd1_G15N_T101L_)_2_ structure, with the donor–strand-complemented PapA subunit (dscPapA) in purple and the donor–strand-exchanged PapA subunit (dsePapA) in orange. The core sheet structure of dsePapA is in a closer conformation than that of dscPapA, as the β-strands on each side of the groove of dsePapA are nearer to each other. Also, the “63–74” loop is ordered in dsePapA and not in dscPapA, as this molecule is missing residues 70 to 73 in this region. The truncated Nte of dscPapA (as indicated above, residues 2 to 8 were removed to create PapA_Ntd1_) is clearly visible in the groove of dsePapA from residue 10 (the two first residues of PapA_Ntd1_G15N_T101L_, which are residues 1 and 9 of full-length PapA, were not defined in the electron density). DsePapA is only visible in the electron density from residue 20, as its Nte is not interacting in the groove of another PapA molecule and is thus disordered. Figure 3B shows the surface of dsePapA bound to the truncated Nte of dscPapA (left panel) and that of dscPapA bound to the G_1_ strand of the chaperone (right panel): the PapD G_1_ strand interacts as expected at the P1 to P4 positions in the groove of dscPapA (right panel), the Nte of which interacts in the P2 to P5 pockets in the groove of dsePapA (left panel). It is noticeable in the left panel of Figure 3B that the dsePapA groove is extending beyond the region occupied by the P2 residue of dscPapA Nte. However, the groove of dscPapA is not extending beyond the P1 pocket, due to the disordered “63–74” loop. Modeling of the first nine residues of PapA Nte in the extended groove of dsePapA shows that this extended groove has the right length and shape to accommodate the nine missing residues in the Nte of PapA_Ntd1_ (unpublished data). Thus, the groove of PapA is long enough to accommodate the extended Nte of another PapA molecule.

### Structure Comparisons of the Grooves of dscPapA in PapD/PapA_Ntd1_G15N_ and dsePapA in PapD/(PapA_Ntd1_G15N_T101L_)_2_

**Figure 4:**
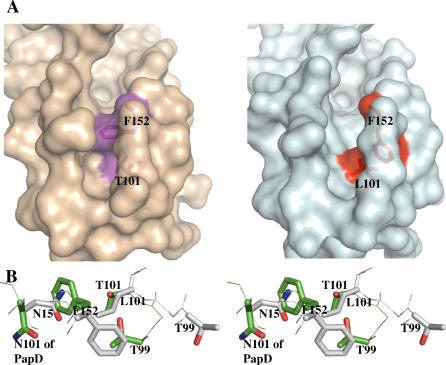
Comparison of PapD/PapA_Ntd1_G15N_ and PapD/PapA_Ntd1_G15N_T101L_ (A) Surface representation of dscPapA in PapD/PapA_Ntd1_G15N_ (left) and dsePapA in PapD/(PapA_Ntd1_G15N_T101L_)_2_ (right). The residues in the groove at the P4 position (F152 [purple and red in left and right panels, respectively]) and the one at the P5 position (T101 [purple] or L101 [red]) are indicated. (B) Stereo view of a superimposition of the residues involved in DSE at the P4 and P5 positions. The green residues are N101 (PapD), F152, T101, and T99 (dscPapA in PapD/PapA_Ntd1_G15N_), whereas the gray residues are N15 (PapA Nte), F152, L101, and T99 (dsePapA in PapD/(PapA_Ntd1_G15N_T101L_)_2_).

Figure 4A shows details of the grooves of dscPapA_Ntd1_G15N_ (left panel) and dsePapA_Ntd1_G15N_T101L_ (right panel). Figure 4A on the left panel shows that in the PapD/PapA_Ntd1_G15N_ structure, the P4 position of the groove is formed by F152. This configuration of F152 is similar to that seen in the equivalent position of the PapD/PapK, PapD/PapE, PapD/PapH, and PapE/K_Nte_ complexes [9,11,18]. As mentioned above, the bulk created in the P4 pocket by F152 and equivalents, by being able to accommodate only a conserved Gly in the Ntes, acts as a registering device that calibrates the positioning of Ntes in the subunits' grooves.

**Figure 5:**
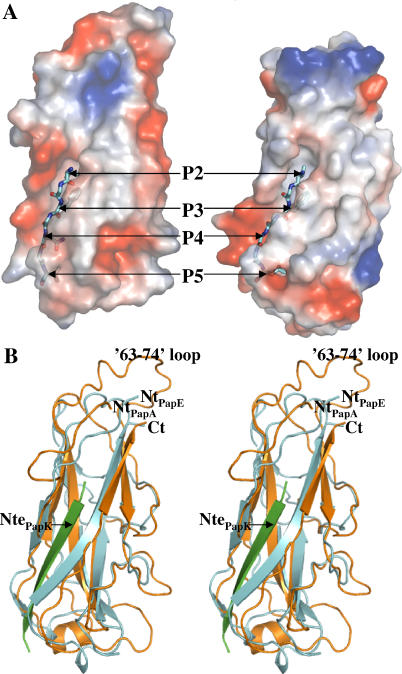
Comparison of dsePapA (in PapD/(PapA_Ntd1_G15N_T101L_)_2_) and dsePapE (in PapE/K_Nte_) (A) Surface representation of dsePapA (left) and dsePapE (right). Residues of PapK_Nte_ as well as dscPapA Nte are shown in stick representation color-coded in red, dark blue, and cyan for oxygen, nitrogen, and carbon atoms, respectively. For clarity, only side chains of the residues interacting in the P2 to P5 pockets are shown. (B) Secondary structure representation of dsePapA (orange) and dsePapE (cyan) bound to K_Nte_ (green). The structures are in stereo ribbon representation. The N- and C-termini of both molecules are indicated, as well as the “63–74” loop in dsePapA.

In contrast to what is observed in the PapE/K_Nte_ or any other chaperone–subunit complex structures, the side chain of F152 has moved out of the P4 pocket of the DsePapA structure (Figure 4A, right panel) and the P4 pocket can now accommodate the N15 mutation (substituted for wild-type G15 in the PapA_Ntd1_G15N_T101L_ mutant to prevent higher-order polymerization). F152 in the dsePapA structure is allowed to move away from the groove position because of the T101L mutation. Indeed, as shown in Figure 4B, the T101L mutation and the insertion of N15 induce a rearrangement of the side-chains in the P4–P5 region, leading to T99 moving out of the P5 region, thereby leaving room for F152 to substitute in its place, the new F152 position being stabilized by L101.

### Structure Comparisons between the Groove of dsePapA in the PapD/(PapA_Ntd1_G15N_T101L_)_2_ Structure and That of dsePapE in the PapE/K_Nte_ Structure

Like dsePapA in the PapD/(PapA_Ntd1_G15N_T101L_)_2_ complex structure, PapE in the PapE/K_Nte_ structure is also donor–strand exchanged (dsePapE), but with the Nte of PapK (K_Nte_) [18]. Comparing dsePapE (Figure 5A, right) with dsePapA (Figure 5A, left) shows that, while the groove of dsePapE stops at the P2 pocket, the groove of dsePapA is extending beyond this pocket. This is due to the presence of the “63–74” loop in dsePapA, a loop which is much shorter in dsePapE, and to the closure of dsePapE groove by PapE N- and C-termini compared with the open groove in dsePapA (Figure 5B).

### Gel Filtration Profiles and EM of the PapA Nte Mutated Constructs

**Figure 6:**
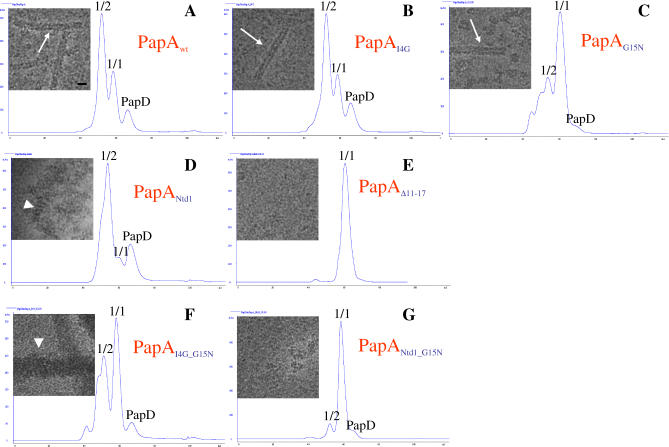
Gel Filtration Profiles and EM pictures of PapD/PapA Wild-Type (wt) and Mutant Complexes Each gel filtration profile of a particular PapA construct is shown with a view of its corresponding EM picture on the left. The complex is labeled on the right. Only the gel filtration peaks corresponding to free PapD, PapD/(PapA)_1_ (1/1) or PapD/(PapA)_2_ (1/2) are labeled. Representative pili are labeled with arrows ([A–C]). Non-pilus polymerization of protein is labeled with arrowheads ([D] and [F]). The scale of all the EM pictures is represented by the black bar on the PapD/PapA_wt_ picture and corresponds to 100 Å.

A number of PapA Nte mutants were next produced in order to evaluate the effect of these mutations on polymerization and pilus formation. In addition to PapA wild-type, six constructs were studied. In the DSE region, a G15N mutation and a Δ11–17 deletion (where the entire DSE region is removed) were made. In the region N-terminal to the DSE region of the Nte, an I4G single-site mutant and the Ntd1 deletion described above were studied. I4 is a bulky residue in that region of the Nte and thus would contribute significantly to the interface, were it to be involved in groove/Nte interaction (see [21] for consideration regarding surface area contribution of residues in the Nte). We also combined the Ntd1 (Δ2–8) deletion with the G15N mutation and the I4G and G15N mutations. Polymerization was assessed by gel filtration immediately after purification of the corresponding PapD/PapA complexes, and pilus formation was assessed after freeze–thaw of PapD/PapA complex preparations using electron microscopy (EM). Thus, gel filtration provides information on the limited polymerization events taking place early on during polymerization, while freeze–thaw of PapD/PapA complexes followed by analysis by EM provides information on the ability of the various PapA molecules to form pili. Results obtained for each of the PapA constructs are presented in Figure 6, where each panel provides the elution profile and EM micrograph for each wild-type and mutated PapA. For those complexes that exhibited a gel filtration peak corresponding to PapD/(PapA)_2_, further polymerization was checked by pooling the fractions corresponding to PapD/(PapA)_2_ and running another gel filtration the next day. For those complexes that did not exhibit polymerization, the PapD/(PapA)_1_ peak was rerun on gel filtration to make sure that indeed no polymers were formed.

Gel filtration of PapD/PapA_wt_ complexes reveals a major 1:2 PapD/PapA (PapD/(PapA)_2_) complex. Yet, this complex polymerizes, as (1) rerunning a gel filtration on the 1:2 complex 24 hours later results in higher order polymers being formed (unpublished data), and (2) pili are formed readily (Figure 6A). These results confirm the existence of a rate-limiting step in the DSE reaction whereby PapD/(PapA)_2_ complex formation appears to be required before DSE can proceed to higher-order polymer forms [23]. The reason for such a rate-limiting step is unclear; it may be that this provides a checkpoint mechanism before committing to full biogenesis of the rod. The PapD/PapA_I4G_ behaves very much like the PapD/PapA_wt_ in the gel filtration, but fewer pili appear to be made (Figure 6B). The single G15N mutation in the DSE region appears to affect polymerization with the detection of 1:3 and 1:4 PapD/PapA complexes (PapD/(PapA)_3_ and PapD/(PapA)_4_), and while pilus formation does occur, the diameters of the pili and their central channels are increased (Figure 6C). Thus, these mutations appear to slow down the reaction in such a way that polymer intermediates are observed after 24 h, but overall, these mutations do not seem to affect the process so stringently that it is unable to proceed to completion. More important is the effect of deleting the region preceding the DSE region (PapD/PapA_Ntd1_; Figure 6D) or deleting the DSE region (PapD/PapA_Δ11–17_; Figure 6E). Neither of these mutants produces pili. Also, while PapD/PapA_Ntd1_ appears to be able to form 1:2 PapD/PapA complexes and aggregates of protein are visible by EM, the PapD/PapA_Δ11–17_ mutant is totally impaired. Thus, both regions (the DSE region and the region N-terminal to it) are important for PapA pilus formation, with the deletion of each of the regions blocking the process at two different stages of PapA polymerization. Combining the DSE (G15N) and non-DSE (I4G) mutations (Figure 6F), or combining the non-DSE deletion (Ntd1) and the single-site DSE (G15N) mutation (Figure 6G) lead to results that confirm our conclusion that both regions of the Nte are important for Pap polymerization and pilus formation. For the combined PapA_I4G_G15N_, large, linear aggregates are found, which are not pili. Higher-order limited polymers (PapD/(PapA)_2_, PapD/(PapA)_3_, and PapD/(PapA)_4_) are observed, showing that this double mutant is not totally impaired, while PapA_Ntd1_G15N_ appears to be severely impaired.

### Conclusions

In this report, we solved the structures of the PapA subunit, the major subunit of the P pilus, before and after DSE, and, based on these structures, we have examined the roles that the various regions in the Nte play in polymerization and pilus formation. We show that polymerization of wild-type PapA transitions through a 1:2 PapD/PapA complex (PapD/(PapA)_2_), and that a triple alteration combining a deletion of the residues preceding the DSE region of the Nte (Ntd1), a mutation of a conserved Gly residue in the DSE region (G15N), and a mutation in the P5 pocket (T101L) stabilizes the PapD/(PapA)_2_ intermediate. The Ntd1 and G15N mutations, individually, do not appear to block the formation of the PapD/(PapA)_2_ intermediate complex, nor do they block formation of higher-order complexes (Figure 6C and 6D). However, the combined Ntd1 and G15N mutations severely impair formation of these complexes (Figure 6G). Thus, the T101L mutation appears to attenuate the severity of the combined Ntd1 and G15N mutations and stabilizes the PapD/(PapA)_2_ intermediate. This may be because partial filling of the P5 pocket by Leu alters the DSE reaction, resulting in increased PapD/(PapA)_2_ formation but abrogating further polymerization events.

The crystal structure of the PapD/(PapA_Ntd1_G15N_T101L_)_2_ suggests that the groove of PapA is longer than the groove of any other Pap subunits of known structure, and that this is why it can accommodate a longer Nte. This led us to suggest that both the DSE region and the region N-terminal to it are important for pilus formation. Site-directed and deletion mutagenesis confirm this view and thus validate earlier published observations by Mu et al. [22], which emphasized the role of the non-DSE region of the Nte. Thus, PapA uses an extended Nte and in that respect appears to be very similar to other major pilus subunits such as SafA of *Salmonella* or Caf1 of *Yersinia* [19,21]. The Nte of SafA, for example, is 17 residues long, and one residue outside its DSE region, F3, was shown to be important in capping the process of DSE and driving the reaction to completion (the DSE region of SafA consists of residues 11 to 17). Indeed, a mutation of F3 to Ala in SafA results in an equilibrium between reaction species because in this mutant, DSE is allowed to proceed in reverse. A possible equivalent of F3 in PapA is I4. Indeed, DSE is somewhat affected by the I4G mutation. Thus, some features common to the assembly of all major pilus subunits are emerging, which include the involvement of an extended protein–protein interface and a potential capping mechanism driving polymer formation to completion. The PapA polymer is however different from the SafA or Caf1 polymer in that it adopts a distinct tertiary superhelical structure. The structures presented here do not provide any clues as to how such a ternary structure could form. Indeed, packing interfaces observed in both the PapD/PapA_Ntd1_G15N_ and PapD/(PapA_Ntd1_G15N_T101L_)_2_ crystals appear irrelevant (unpublished data). However, the structure of PapA elucidated here provides the basis for complementing the work by Mu et al. [24] and characterizing further the PapA/PapA interactions that preside over superhelix formation.

## Materials and Methods

### Plasmid preparations.

See Text S1.

### Protein expression and purification of the PapD_His_/PapA complexes.

The eight PapD_His_/PapA constructs (PapD_His_/PapA_wt_, PapD_His_/PapA_Ntd1_, PapD_His_/PapA_Δ11–17_, PapD_His_/PapA_G15N_, PapD_His_/PapA_I4G_, PapD_His_/PapA_Ntd1_G15N_, PapD_His_/PapA_I4G_G15N_, and PapD_His_/PapA_Ntd1_G15N_T101L_) were transformed one at a time into E. coli C600 cells and grown in a 5-l fermentor vessel containing Terrific Broth (TB; Sigma, http://www.sigmaaldrich.com) kept at 37 °C and shaken at 600 rpm. The cells were induced with 1 mM IPTG, once the OD_600_ reached a value of 0.9, and kept growing for another 3.5 h. The complexes were purified after periplasmic extraction using Cobalt-affinity chromatography (Talon; Clontech, http://www.clontech.com), followed by hydrophobic interaction chromatography (phenyl source; GE Healthcare, http://www.gehealthcare.com), ending with a gel filtration step in 20 mM TrisHCl (pH 7.5) and 20 mM NaCl, using a Superdex75 120 ml column. This last step was crucial for separating the different polymer forms of PapA in complex with PapD (PapD/(PapA)_1_, PapD/(PapA)_2_, PapD/(PapA)_3_…).

### Purification of PapD_His_/(PapA_Ntd1_G15N_T101L_)_2_.

The PapD_His_/(PapA_Ntd1_G15N_T101L_)_2_ complex was purified as explained above. However after the gel filtration step, there was a second major peak that eluted at a volume of around 54 ml (the 1:1 complex eluted at around 60 ml) and was interpreted as a 1:2 complex (PapD/(PapA)_2_). The 1:2 complex was concentrated to 13 mg/ml for crystallization trials.

### Purification of PapD/PapA_Ntd1_G15N_.

The PapD/PapA_Ntd1_G15N_ complex was purified from periplasmic extracts using cation-exchange chromatography (SP HiTrap HP column; GE Healthcare) followed by hydrophobic interaction chromatography (phenyl source). The purification was completed by a gel filtration step in 20 mM MES (pH 6.0) and 20 mM NaCl on a Superdex75 120 ml column. The 1:1 complex eluted at a volume of around 60 ml and was concentrated to 8 mg/ml for crystallization trials.

### In vitro DSE experiments.

See Text S1.

### PapD/PapA_Ntd1_G15N_ and PapD_His_/(PapA_Ntd1_G15N_T101L_)_2_ crystallization and structure determination.

*PapD/PapA_Ntd1_G15N_:* two crystal forms of the complex were obtained; in both cases PapD/PapA_Ntd1_G15N_ was crystallized at room temperature in a hanging drop. In the first crystal form (plates), the drop was equilibrated against a reservoir solution containing 25% PEG8K, 10% isopropanol, and 0.1 M MES (pH 6.6). In the second crystal form (rods), the drop was equilibrated against a reservoir solution containing 2 M ammonium sulfate and 0.1 M Na acetate (pH 5.6). The plates belonged to space group C2 and diffracted to 3.2 Å, whereas the rods belonged to space group P3_2_21 with cell dimensions a = 167 Å, b = 167 Å, c = 178 Å, and diffracted to 2.6 Å The solvent content is 71%, with 4 PapD/PapA_Ntd1_G15N_ complexes per asymmetric unit (Table S2). The data from one single rod-shaped crystal was processed to 2.6 Å, and the structure solved by molecular replacement using PapD/PapK as a search model with the program AMoRe [25]. The PapK molecule was first modeled to a poly-alanine chain prior to refinement. The first refinements were performed using simulated annealing and noncrystallographic symmetry restraints for the four complexes in the asymmetric unit, using CNS [26]. Then successive cycles of manual rebuilding with O [27] and conjugate gradient minimization with CNS were performed. B factors were refined individually. Toward the end of the refinement, the noncrystallographic symmetry restraints were only applied to some parts of the β-sheet core of the complexes. The refinement converged to the final R values of R = 23.0% and Rfree = 26.6% with good stereochemistry.

*PapD_His_/(PapA_Ntd1_G15N_T101L_)_2_:* This complex was crystallized at room temperature in a hanging drop equilibrated against a reservoir solution containing 12% PEG8K, 5% isopropanol, and 0.1 M TrisHCl (pH 7.5). These plates belonged to space group C2, with one complex per asymmetric unit (62% solvent) and were improved by microseeding. The cell dimensions are a = 133 Å, b = 74 Å, c = 80 Å, and β= 109°. The data from one plate was processed to a resolution of 2.5 Å. The structure was solved by molecular replacement (AMoRe) using the previously solved PapD/PapA_Ntd1_G15N_ structure as a search model. In the electron density map, there was extra density near the N-terminal extension of the PapA molecule. This density was good enough to enable manual fitting of another PapA molecule next to the first one. Then successive cycles of conjugate gradient minimization with CNS and manual rebuilding with O (http://xray.bmc.uu.se/alwyn) enabled rebuilding of some of the second PapA molecule loops that differed from the first one. B factors were refined individually, and no noncrystallographic symmetry restraint was applied between the two PapA molecules. The refinement converged to the final values of R = 22.3% and Rfree = 26.2% with good stereochemistry (Table S2).

### Electron microscopy of the PapD/PapA complexes.

PapD/PapA (chaperone/pilin) samples of wild-type and mutant PapA proteins were frozen and thawed five times in liquid nitrogen at 150 μg/ml in 10–20 mM TrisHCl (pH 7.6) and 20–50 mM NaCl. The frozen–thawed sample was used either on the same day, or allowed to sit at 4 °C for as long as 40 d to enhance oligomerization of PapA subunits. The sample (5 μl) was placed on carbon-coated, glow-discharged grids, washed with 10 mM TrisHCl (pH 7.6), negatively stained with 1% uranyl acetate, and imaged on a Philips CM12 electron microscope (no longer available).

## Supporting Information

Figure S1 Sequence Alignment of Pap Subunit Ntes The sequences have been aligned using the conserved Glycine (pink boxes) and the alternating hydrophobic residues of the DSE region (indicated). The P2 to P5 residues (yellow boxes) of the Nte that insert into the P2 to P5 regions (or pocket) of the subunit grooves are indicated. The C-terminal borders of the Ntd1 and Δ11–17 deletions are indicated.
(123 KB PPT)

Figure S2 Biochemical Properties of Mutants Used for Crystallization and Structure Determination (A) Polymerization of PapD/PapA_G15N_ (upper panel), PapD/PapA_Ntd1_G15N_ (middle panel), and PapD/PapA_Ntd1_G15N_T101L_. The gel filtration elution profile is shown in blue. Each panel is labeled in red according to the form of PapA used to generate the corresponding results. The peaks corresponding to PapD, to a 1:1 ratio of PapD/PapA (PapD/(PapA)_1_), and to a 1:2 ratio of PapD/(PapA)_2_ are indicated as PapD, 1/1, and 1/2, respectively.
(B) In vitro DSE experiments showing the PAGE analysis results from the nickel-NTA spin columns after the complexes had been left overnight at 24 °C in the presence of the Nte_PapA_ peptide. Left part of the gel: molecular weight markers, eluate (el), flow through sample with no peptide (0), and flow through samples with increasing molar ratios 1, 10, 25, and 50 of Nte_PapA_ peptide added to the PapD_His_/PapA_Ntd1_G15N_ complex. Right part of the gel: similar experiment with the PapD_His_/PapA_Ntd1_G15N_T101L_ complex. The bands corresponding to PapD_His_ and the PapA mutants are indicated.
(182 KB PPT)

Table S1 Oligonucleotides Used in this Study For the PapD_HIS_, PapA_Ntd1_, and PapA_Δ11_17_ constructs, primer 5′ ends were phosphorylated.
(51 KB DOC)

Table S2 Data Collection and Refinement Statistics (45 KB DOC)

Text S1 Materials and Methods Discussion includes plasmid preparations and in vitro DSE experiments.
(50 KB DOC)

### Accession Numbers

The Protein Data Bank (http://www.rcsb.org/pdb) accession numbers for the coordinates for the structures of the complexes mentioned in this article are PapD/PapA_Ntd1_G15N_ (2uy7), PapD_His_/(PapA_Ntd1_G15N_T101L_)_2_ (2uy6), PapE/K_Nte_ (1N12), and PapD/PapK (1PDK).

## Abbreviations

DSC: donor–strand complementation
dscPapA: donor–strand-complemented PapA subunit
DSE: donor–strand exchange
dsePapA: donor–strand-exchanged PapA subunit
EM: electron microscopy
Nte: N-terminal extension
