# Supplementary material for: Crystal Structure of the P Pilus Rod Subunit PapA
Source: PLoS Pathog. 2007 May 18;3(5):e73. doi: 10.1371/journal.ppat.0030073 (PMC1868955; doi:10.1371/journal.ppat.0030073)
Supplement: Supplementary file 1 [file ppat.0030073.sd001.doc]

**Supplemental Materials**

**Materials and Methods**

**Plasmid preparations.** For all the PapD/PapA (wild type and mutants) complexes, the PapD gene was cloned by PCR from *E. coli* UTI89 genomic DNA using oligonucleotides flanked by BamHI and XbaI restriction sites (Table 1 of supplementary materials). The PapA gene was extracted from plasmid pMMB66 using EcoRI and BamHI restriction enzymes [1]. The PapA and PapD genes were introduced into a single pTrc99A plasmid by a 3 point ligation resulting in pTRC-DA. In order to design the Ntd1 (missing residues 2 to 8) or Δ11-17 (missing residues 11 to 17) mutants of PapA, a PCR of the above pTRC-DA plasmid was carried out using 5’ phosphorylated primers flanking the region to be deleted (residues 2 to 8 for PapA_Ntd1_ or residues 11 to 17 for PapA_Δ11_17_) followed by ligation of the amplified product (Table 1 of supplementary materials), producing pTRC-DA_Ntd1_ and pTRC-DA_Δ11-17_ respectively. The mutants: PapD/PapA_G15N_ and PapD/PapA_I4G_ were made from the pTRC-DA, PapD/PapA_Ntd1_G15N_ was made from PapD/PapA_Ntd1_, PapD/PapA_I4G_G15N_ was made from PapD/PapA_G15N_ and PapD/PapA_Ntd1_G15N_T101L_ from PapD/PapA_Ntd1_G15N_. All these point mutations were made using the Strategene QuickChange Mutagenesis Kit (Table 1 of supplementary materials). In order to design the PapD His tagged version of all the plasmids above, these constructs were modified using 5’ phosphorylated primers containing the coding sequence for 6 histidines plus a short linker region (Ser-Gly; Table 1 of supplementary materials).

***In vitro* donor strand exchange (DSE) experiments.** For each DSE experiment, 50 μg of complex (PapD_His_/PapA_Ntd1_G15N_ or PapD_His_/PapA_Ntd1_G15N_T101L_) were mixed with the Nte_PapA_ N-terminal extension peptide (sequence: Ac-APTIPQGQGKVTFNGTVVD-NH2 obtained from QCB) in a peptide/complex molar ratio of 0 (control), 1, 10, 25 and 50 in 50mM Tris-HCl pH7.5 and 50mM NaCl (Buffer A). The mixtures were then incubated at 24ºC in a shaker (150 rpm) overnight. After incubation, 100 μl (50 μg) of the mixture were loaded onto the Nickel-NTA spin columns pre-washed with buffer A. The columns were then centrifuged for 2 minutes at 700 g and the flow-through samples reloaded two more times. The flow-throughs were then analyzed using SDS-PAGE.

**References**

1. Lee YM, DiGiuseppe PA, Silhavy TJ, Hultgren SJ (2004) P pilus assembly motif necessary for activation of the CpxRA pathway by PapE in Escherichia coli. J Bacteriol 186: 4326-37.
