# Supplementary material for: Crystal Structure of the P Pilus Rod Subunit PapA
Source: PLoS Pathog. 2007 May 18;3(5):e73. doi: 10.1371/journal.ppat.0030073 (PMC1868955; doi:10.1371/journal.ppat.0030073)
Supplement: Supplementary file 2 [file ppat.0030073.st001.doc]

**Supplemental Materials**

**Table S1.** Oligonucleotides used in this study. For the PapD_HIS_, PapA_Ntd1_ and PapA_Δ11_17_ constructs, primers 5’ ends were phosphorylated.

| Construct | Forward primer | Reverse primer |
| --- | --- | --- |
| PapD | CGC GGA TCC CGG CTG ATG AAT GCG GTT ATG CC | CTA GTC TAG ACA CAA CCA TAA TAT CCC ACC CGC |
| PapD_HIS_ | CAT CAC CAT CAC TAA TGT ACC GCA ATA ACG GTT AAA TGC GGG | GTG ATG CGA TCC TTT CTC TTT TTT CAC AGA GCA ACG GCT GCC |
| PapA_Ntd1_ | GGT AAA GTA ACT TTT AAC GGA ACT GTT G | AGC AGC ATT ATT TAC ACC AAA AGA CAC |
| PapA_Δ11_17_ | GTT GAT GCT CCA TGC AGC ATT TCT CAG | TTT ACC CTG CCC CTG TGG AAT AGT TGG |
| PapA_G15N_ | GGG TAA AGT AAC TTT TAA CAA CAC TGT TGT TGA TGC TCC | GGA GCA TCA ACA ACA GTG TTG TTA AAA GTT ACT TTA CCC |
| PapA_I4G_ | AAT AAT GCT GCT CCA ACT GGT CCA CAG GGG CAG GG | ACC CTG CCC CTG TGG ACC AGT TGG AGC AGC ATT ATT TAC |
| PapA_Ntd1_G15N_ | GGT AAA GTA ACT TTT AAC AAC ACT GTT GTT GAT GCT CC | GGA GCA TCA ACA ACA GTG TTG TTA AAA GTT ACT TTA CC |
| PapA_I4G_G15N_ | AAT AAT GCT GCT CCA ACT GGT CCA CAG GGG CAG GG | ACC CTG CCC CTG TGG ACC AGT TGG AGC AGC ATT ATT TAC |
| PapA_Ntd1_G15N_T101L_ | GAT ACA AAT GGT GGT ACG GGC CTG GCT ATC GTA GTT CAG GGG | CTG AAC TAC GAT AGC CAG GCC CGT ACC ACC ATT TGT ATC TAG C |
