# Supplementary material for: Crystal Structure of the P Pilus Rod Subunit PapA
Source: PLoS Pathog. 2007 May 18;3(5):e73. doi: 10.1371/journal.ppat.0030073 (PMC1868955; doi:10.1371/journal.ppat.0030073)
Supplement: Supplementary file 3 [file ppat.0030073.st002.doc]

**Supplemental Materials**

**Table S2.** Data Collection and Refinement Statistics

| *Data collection* |  | |  | |
| --- | --- | --- | --- | --- |
| Data set  (ESRF ID29) | PapD/PapA_Ntd1_G15N_ | | PapD_HIS_/(PapA_Ntd1_G15N_T101L_)x2 | |
| Resolution | 75.8-2.6 Å | | 76.3-2.5 Å | |
| Total/Unique  Reflections | 455,235/88,336 | | 174,597/25,542 | |
| Completeness ^1^(%) | 99.8 (99.7) | | 100.0 (99.5) | |
| R_sym_(%)^2^ | 0.09 (0.447) | | 0.092 (0.382) | |
| I/σ(I)^3^ | 7.3 (1.7) | | 6.2 (1.9) | |
|  |  | |  | |
| *Refinement* |  | |  | |
| Resolution (Å) | 75.8-2.6 | | 76.3-2.5 | |
| Number of reflections^4^ | 88,123 | | 25,532 | |
| Total number of atoms^5^ | 11337 | | 3897 | |
| R/R_free_ (%)^6^ | 23.0/26.6 | | 22.3/26.1 | |
| r.m.s. deviations^7^ | Bonds (Å)  0.007 | | Bonds (Å)  0.006 | |
|  | Angles (°)  1.35 | | Angles (°)  1.30 | |
|  | B-values (Å^2^) | | B-values (Å^2^) | |
|  | main chain | side chain | main chain | side chain |
|  | 1.45 | 2.0 | 1.40 | 2.0 |

^1^Completeness for I/σ(I) >0, high resolution shell in parentheses.

^2^R_sym_ =∑|I-〈I〉|∑I, where I=observed intensity and 〈I〉=average intensity from multiple observations of symmetry related reflections; high resolution shell in parentheses.

^3^I/σ(I) in high resolution shell in parentheses.

^4^Number of reflections (F/σ(F) > 0.0) in the “working” set.

^5^Includes water molecules and sulfate ions

^6^R_free_ was calculated on the basis of 5% of the total number of reflections randomly omitted from the refinement.

^7^Deviations from ideal bond lengths and angles and in B factors of bonded atoms
